# Chemically-Gated and Sustained Molecular Transport through Nanoporous Gold Thin Films in Biofouling Conditions

**DOI:** 10.3390/nano11020498

**Published:** 2021-02-16

**Authors:** Barath Palanisamy, Noah Goshi, Erkin Seker

**Affiliations:** 1 Department of Biomedical Engineering, University of California, Davis, CA 95616, USA; bpalanisamy@ucdavis.edu (B.P.); nkgoshi@ucdavis.edu (N.G.); 2 Department of Electrical and Computer Engineering, University of California, Davis, CA 95616, USA

**Keywords:** drug delivery, stimulus-responsive, nanoporous gold, sustained release, biofouling

## Abstract

Sustained release and replenishment of the drug depot are essential for the long-term functionality of implantable drug-delivery devices. This study demonstrates the use nanoporous gold (np-Au) thin films for in-plane transport of fluorescein (a small-molecule drug surrogate) over large (mm-scale) distances from a distal reservoir to the site of delivery, thereby establishing a constant flux of molecular release. In the absence of halides, the fluorescein transport is negligible due to a strong non-specific interaction of fluorescein with the pore walls. However, in the presence of physiologically relevant concentration of ions, halides preferentially adsorb onto the gold surface, minimizing the fluorescein–gold interactions and thus enabling in-plane fluorescein transport. In addition, the nanoporous film serves as an intrinsic size-exclusion matrix and allows for sustained release in biofouling conditions (dilute serum). The molecular release is reproducibly controlled by gating it in response to the presence of halides at the reservoir (source) and the release site (sink) without external triggers (e.g., electrical and mechanical).

## 1. Introduction

Implantable drug delivery platforms are emerging as powerful technologies for treating and managing a wide range of medical conditions, including cancer, diabetes, and epilepsy [1,2,3,4,5,6,7]. These drug delivery modalities have shown promise in overcoming several pharmaceutical challenges by improving permeation across the blood–brain barrier [8], reducing systemic side effects through targeted delivery at the site of interest [9], and maintaining the delivery dose within the therapeutic window for high efficacy and low toxicity [10]. One class of drug delivery technology is based on the use of macro-scale infusion pumps or miniaturized microelectromechanical systems (MEMS)-based pumps, where the delivery segment (e.g., cannula) and the reservoir can be both implanted [11,12,13]. In both cases, the pharmaceuticals are delivered in a liquid vehicle solution by convective means, which leads to the problem of potentially damaging increase of local pressure in the target tissue. Ideally, only the pharmaceutical molecules should be delivered into the tissue, which requires a non-convective transport mechanism. To that end, non-degrading nanostructured materials (e.g., porous anodic alumina [14], carbon nanotubes [15,16], and porous silicon [1,17]) and degrading polymers [18,19] have shown promise for molecular release of the pharmaceuticals without volume or pressure change at the delivery site. However, there are still obstacles, such as biofouling of the nanostructured material, controlling the release rate, and the unavoidable depletion of drug molecules in a fully implanted system. While there have been some remedies (e.g., antibiofouling coatings [20] and size-exclusion membranes [21]) and stimulus-responsive gating mechanisms [22,23,24,25] for the former two obstacles, the latter has remained non-trivial, especially for non-convective drug delivery platforms. There have been creative solutions, such as replenishment of drug depot via selective capture of pharmaceuticals at the delivery site [26]. However, these solutions still have shortcomings such as their applicability to anatomical sites with reduced transport from the blood (e.g., central nervous system).

Taken together, implantable drug delivery platforms that rely on non-convective transport require the ability to (i) sustain pharmaceutical release, (ii) allow for pharmaceutical replenishment at a location distal to the delivery site, (ii) maintain functionality in biofouling conditions, and (iii) regulate release rate in response to external and internal stimuli. In this paper, we use nanoporous gold (np-Au) to address these challenges. Np-Au is fabricated through the dissolution of silver from a silver-rich gold-silver alloy where surface diffusion of gold at the metal-electrolyte interface creates a bicontinuous open-pore structure [27]. The morphology can be tuned by post-processing techniques (e.g., thermal [28,29,30], electrical [31], electrochemical [32], and photo-thermal [33,34]) to yield np-Au with ligament and pore diameters spanning several orders of magnitudes (10–1000 s nm) with self-similar morphologies [28,35]. In addition to the tunable morphology, np-Au exhibits other desirable features such as high surface area to volume ratio [36], electrical conductivity [37], biocompatibility [38], biofouling resilience [39], compatibility with conventional microfabrication processes [40,41], and facile surface functionalization via gold-thiol chemistry [42]. These benefits make np-Au an attractive material system for drug delivery [43], short nucleic acid detection [44], and catalysis [45,46]. Here, we report a computational and experimental study of in-plane molecular transport in np-Au thin films integrated into a microfluidic model of a reservoir distal to the pharmaceutical delivery site. The study demonstrates sustained (via replenishment of molecules at the distal reservoir) and controllable (via gating with physiologically-relevant solutions) molecular release in biofouling conditions (dilute fetal bovine serum).

## 2. Materials and Methods

### 2.1. Fabrication and Characterization of np-Au Thin Films

**Figure 1:**
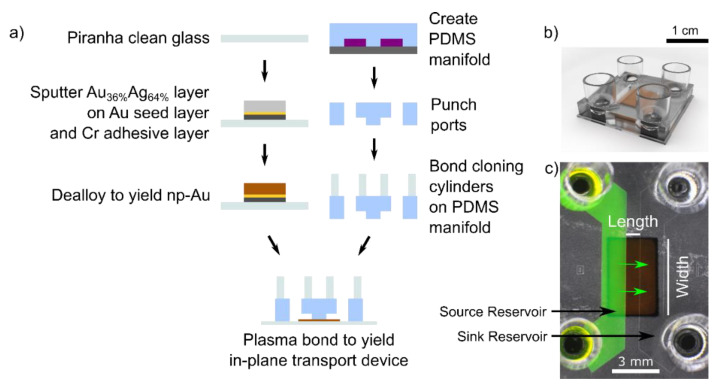
(**a**) Fabrication schematic of the in-plane transport device. (**b**) A 3D rendering of the device illustrating the fully fabricated device along with the four cloning cylinders on top of the PDMS manifold. (**c**) Overhead view of the in-plane transport device, with 100 µM of sodium fluorescein in the source reservoir and the matching vehicle solution in the sink reservoir.

Np-Au thin films were fabricated as previously described [47]. Briefly, a stencil mask was used to pattern np-Au and pl-Au thin films on piranha-cleaned glass coverslips (Figure 1a). For np-Au, Au_0.36_Ag_0.64_ (atomic %) alloy was deposited on top of Cr adhesion layer (160 nm-thick) and Au seed layer (80 nm-thick) via sputter-deposition. In order to fabricate different thicknesses of np-Au thin films, the sputter-deposition time of the alloy was varied (10 min for 500 nm-nominal thickness for np-Au, 5 min for 250 nm nominal thickness for t-np-Au). The alloy-coated coverslips were then dealloyed in nitric acid (70%) at 55 °C for 15 min to produce the as dealloyed np-Au film. To obtain the coarser morphology (A-np-Au), a subset of the np-Au thin films were thermally treated on a hotplate at 250 °C for 40 s. Planar gold (pl-Au) films were fabricated by sputtering only Au (10 min) over the Cr adhesion layer with no dealloying step. Scanning electron microscopy (SEM) was used to obtain high-resolution images of top and side views of each np-Au thin film type at 100,000× magnification (FEI Nova Nano-SEM430, Phenom World, Hillsboro, OR, USA). ImageJ was used to analyze each film’s thickness and surface pore coverage using previously described methods [47].

### 2.2. Transport Characterization

The construction of the in-plane transport device followed standard microfluidic and soft lithography techniques. Using photolithography, a silicon wafer mold with SU-8 (75 µm-thick) features with a source reservoir and a sink reservoir was fabricated. A 10:1 mass ratio of elastomer base and curing agent (Dow Corning, Midland, MI, USA) was mixed and poured onto the mold. After removing the air bubbles under vacuum, the mold and elastomer mixture were cured at 80 °C for 2 h to cross-link the polymer. A biopsy punch was used to open holes at the corners of each reservoirs, and cloning cylinders (Sigma-Aldrich, St. Louis, MO, USA) were fixed over the opening using additional PDMS (Figure 1b). The PDMS manifold was permanently bonded over the np-Au or Au patterned thin-film coverslips following treatment with air-plasma at 10 W for 30 s (Harrick Plasma PDC-32G, Ithaca, NY, USA). Depending on the experimental condition, 150 µL of either DI water or Dulbecco’s phosphate buffered saline with MgCl_2_ (0.1 g L^−1^) and CaCl_2_ (0.133 g L^−1^) (PBS) was added to each device’s four cloning cylinders to ensure hydrophilicity and wicking of the solution throughout the thin films. For the devices in the DI water condition, the source reservoir was replaced with 100 µM of sodium fluorescein (Sigma-Aldrich, St. Louis, MO, USA) dissolved in DI water, while for the PBS conditions the source reservoir was replaced with 100 µM fluorescein dissolved in PBS. A top view of this device illustrates the fluorescein solution in the source reservoir and vehicle solution in the sink reservoir (Figure 1c). To quantify the amount of fluorescein accumulating in the sink reservoir, samples (10 µL) from each cloning cylinders on the sink side were sampled every 12 h for 3 days. Following sampling, the same volume of vehicle solution was added maintaining the liquid volume in the sink reservoir. Prior to using a fluorospectrometer (Nanodrop ND-3300, Thermo Fisher Scientific, Waltham, MA, USA) to obtain the fluorescence intensity attributed to fluorescein (emission λ = 515 nm), NaOH (10 µL, 50 mM) was added to each sample to ensure maximum fluorescence. The cumulative release of fluorescein with respect to release duration was obtained by fluorometric quantification of the samples. To verify the experimental results, COMSOL Multiphysics^®^ 5.5 was used to obtain a two-dimensional numerical solution for in-plane transport using the Species Transport in Porous Media module. For the DI water condition, in which the Langmuir isotherm model was used to simulate adsorption phenomenon, we performed through-thickness release experiments (as described previously [43]) to obtain the Langmuir isotherm constant (*K_eq_*) and maximum available adsorption sites (*Q_max_*), two parameters needed for numerical simulation (Figure S1). The geometry was constructed via three tangent rectangles each having a length of 1 mm and height as the thickness for each film (Figure S2a). Left edges and right edges were constrained to 100 µM and 0 µM, respectively. We assumed that at *t* = 0, the initial concentration in the film was 0. We computed solutions from 0 to 3 days with hourly increments. To calculate the total mass of fluorescein released into the sink reservoir, we solved for the flux at x = 2 mm, multiplied it by the cross-sectional area of the thin film normal to the molecular transport direction, and integrated it with respect to time. Cross sectional area was calculated via multiplying film thickness and width (5 mm).

### 2.3. Biofouling Resilience

To determine the influence of the biofouling medium on the transport of fluorescein ions, we repeated the in-plane transport experiments described earlier; however, the source reservoir was filled with 100 µM of sodium fluorescein in PBS and the sink reservoir was filled with either FBS (10%) in PBS or only PBS as the control. The fluorescein concentration in the sink reservoir was quantified every 12 h for 6 days. To account for evaporation, 3 µL of DI was added to each cloning cylinder at each sampling time point and the evaporation/volume additions were taken into account for the concentration calculations.

### 2.4. Chemical Gating

For the gating experiments, we alternated either the source or sink reservoir content with DI water or PBS. For source gating, we switched the source reservoir content from fluorescein in DI water (Fluo + DI) to fluorescein in PBS (Fluo + PBS) while maintaining the sink in DI water. For sink gating, we switched the sink reservoir content from DI water to PBS while maintaining the source reservoir with Fluo + DI. DI water (3 µL) was added to each cloning cylinder to account for evaporation effects. At the time of switching the reservoir contents, the liquid of interest was completed removed and washed three times with DI water. Next, the alternate solution was added to the empty cloning cylinders. The fluorescein concentration in sink reservoir was evaluated every 12 h for 20 days. Since replacing the sink reservoir content removed the fluorescein molecules (resetting the concentration to zero), we only displayed the cumulative release for clarity.

### 2.5. Statistical Analysis

For each experiment, a minimum of three replicates (*n* = 3) was used. For statistical comparison of multiple groups, one-way ANOVA followed by a post-hoc Tukey test was used to compare each film’s morphology, thickness, and transport rates in various gating scenarios (sink gating vs. source gating). A two-tailed Student’s *t*-test assuming unequal variances was performed to compare transport rates in biofouling conditions. For all experiments, the statistical significance was determined at *p* < 0.05.

## 3. Results and Discussion

### 3.1. Nanoporous Gold Thin Film Properties

**Figure 2:**
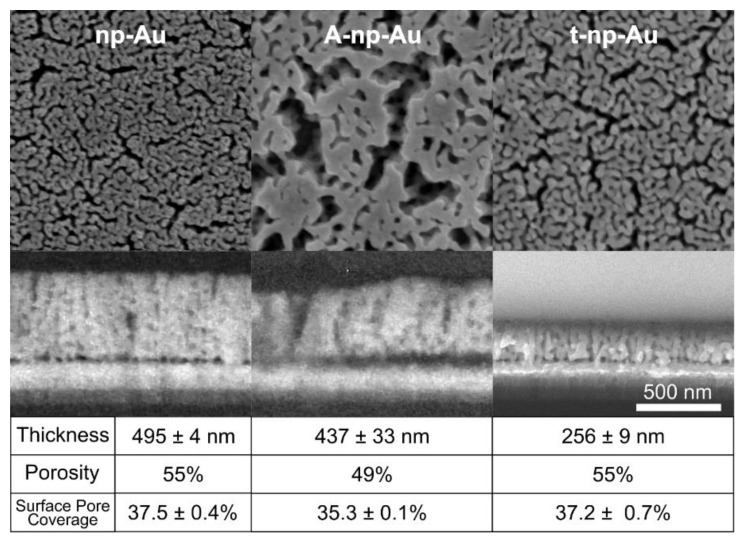
Top- and side-view scanning electron microscope images of various thicknesses and morphologies of gold thin films. The A-np-Au film exhibits a coarser morphology and 11.7% reduction in thickness. The t-np-Au films exhibits similar morphology as np-Au, but with halved thickness. Scale bar applies to all images (mean ± standard deviation, *n* = 3).

We fabricated three types of np-Au thin-film samples by varying the film thickness and pore morphology: np-Au (500 nm nominal thickness; original morphology), annealed np-Au (A-np-Au; 500 nm nominal thickness; coarsened morphology), and thin np-Au (t-np-Au; 250 nm nominal thickness; original morphology). The np-Au films were produced by successive sputter-deposition of a chrome adhesion layer, gold seed layer, and gold-silver precursor alloy, followed by its subsequent dealloying in nitric acid to obtain a bicontinuous open-pore structure, as outlined in Figure 1a. Figure 2 shows the top view and cross-section of the resulting np-Au films and the corresponding thicknesses, volumetric porosity, and surface pore coverage. Thermal treatment enhances surface diffusion of gold and leads to the coarser pore morphology in A-np-Au [28]. During this process, the substrate prevents planar contraction and as a result decreases the thickness by 11.7% [48]. The volumetric porosity (void percentage) of np-Au is primarily based on the initial alloy composition, where the porosity is reduced due to the film shrinkage during dealloying, resulting in a porosity of 55% for both the regular np-Au and the thinner t-np-Au, as described previous [43]. For the A-np-Au sample, the volumetric porosity was reduced to 49% due to film shrinkage during thermal annealing (Table S1). There was no statistically significant difference (*p* value = 0.57) in the surface pore coverage (obtained by image processing) for the two np-Au films of different thicknesses (495 ± 4 nm and 256 ± 9 nm, respectively) in agreement with previous studies [41,43]. The repeatable fabrication approach for creating np-Au thin films with varying thickness and morphology allows for carrying out the systematic studies of molecular transport described next.

### 3.2. In-Plane Fluorescein Transport

As stated earlier, a major obstacle in implantable drug delivery systems is the need to replenish the drug depot for continuous device functionality [49]. Common nanoporous materials for drug delivery, such as porous anodic alumina [14,50] or track-etched membranes [51,52] have columnar pores without lateral connectivity. In contrast, np-Au thin films have lateral pore connectivity that should allow for in-plane molecular transport over significant distances [53], such as between a refillable, distal drug reservoir and the actual site of drug release (Figure 1c). Here, we investigated this functionality, particularly with a focus on the influence of film properties and ionic strength at the reservoir and release sites.

**Figure 3:**
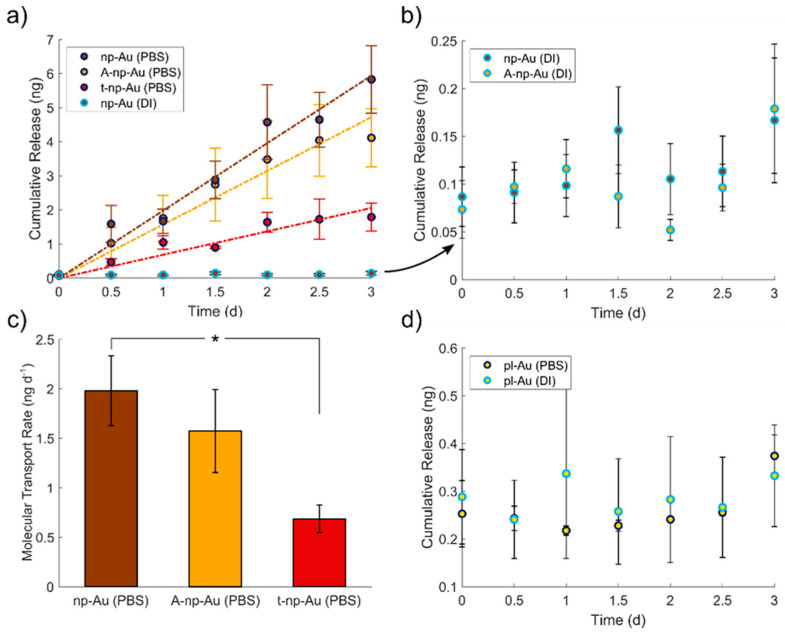
Comparing the in-plane transport through np-Au thin-films under varying conditions. (**a**,**b**) Cumulative release of fluorescein through the np-Au thin-films, indicating a linear increase in cumulative release (indicative of a steady-state transport rate) for all PBS conditions, while minimal transport is observed in DI water (mean ± SE, *n* = 4 for all PBS conditions and *n* = 6 for all DI water conditions). (**c**) Comparing the transport rate of fluorescein through each of the np-Au thin-film types in PBS. Asterisks indicate statistically significant groups (*p* < 0.05). (**d**) Planar gold (pl-Au) control sample in both DI and PBS conditions did not show any molecular transport (mean ± SE, *n* = 3).

We first investigated in-plane transport of fluorescein ions through np-Au thin films under different ionic environments. Fluorescein is a common surrogate for small-molecule drugs, such as cytosine arabinoside (Ara-C) [54], and its intrinsic fluorescence allows for easy characterization via fluorometric methods. As a model test system to represent a distal reservoir (source) and drug release site (sink) connected via an np-Au thin film, we fabricated polydimethylsiloxane (PDMS) chambers by soft lithography and permanently bonded the PDMS manifold over the np-Au thin-film test structures (Figure 1a,b). The source reservoir was filled with 100 µM sodium fluorescein in either deionized (DI) water or Dulbecco’s phosphate buffered saline with MgCl_2_ (0.10 g L^−1^) and CaCl_2_ (0.13 g L^−1^) (PBS) and the matching vehicle solution (DI water or PBS) was added to the sink reservoir (Figure 1c). The sink reservoirs were sampled every 12 h for three days and evaluated with a fluorospectrometer to capture the temporal profiles of fluorescein transport through the np-Au thin film. After 72 h, the PBS condition for all np-Au film types showed noticeable fluorescein transport through the np-Au, while no transport was detected for any of the DI water conditions (Figure 3a,b). We attributed this observation to the influence of halides on molecular transport in np-Au thin films. Specifically, in the absence of halides, fluorescein molecules non-specifically adsorb onto the exposed gold surfaces in the pores via van der Waals interactions and drastically hinder the transport of fluorescein and its release into the sink reservoir. This is in agreement with our findings from fluorescein release experiments from submicron-thick np-Au films, where the addition of halides with higher affinity to gold displaced the non-specifically adsorbed fluorescein molecules and resulted in burst release of fluorescein from the thin films [55]. It should be noted that while there was still fluorescein release for the through-thickness transport (submicron transport distance) in DI water, the significantly longer transport distance (1 mm) for in-plane transport essentially ceased any fluorescein release in this study. Unlike for through-thickness release, the coarser films (A-np-Au) did not produce a significant difference in the transport profile in DI water (Figure 3b), suggesting that the increased pore size plays a minor role on fluorescein transport rate over these long distances. Regardless of the film type, the presence of PBS resulted in the sustained fluorescein release into the sink reservoir, as illustrated by the linear cumulative release (Figure 3a). This sustained release is indicative of sustained molecular in-plane transport and is again due to competitive halide adsorption to the gold surface that enhances in-plane transport of fluorescein molecules. 

To mathematically support the putative underlying adsorption-based mechanism, we utilized the extended Langmuir isotherm model based on each molecular species’ magnitude of adsorption [56]. This model can be used to predict the competitive adsorption of multiple species onto a single substrate, and has been used to model adsorption of acidic dyes on activated carbon [57,58,59]. The pair of equations is given as

(1) $$\theta_{f}=\frac{K_{eq,f}C_{f}}{1+K_{eq,Cl}C_{Cl}+K_{eq,f}C_{f}}\;\theta_{Cl}=\frac{K_{eq,Cl}C_{Cl}}{1+K_{eq,Cl}C_{Cl}+K_{eq,f}C_{f}}$$

where *θ* is the ratio of adsorbed ions and available binding sites, *C* is the concentration of ions in the pores, and *K_eq_* is the Langmuir constant. The concentration of fluorescein ions in the source reservoir was 100 µM. The concentration of chloride ions in PBS is 142 mM. *K_eq_* for each fluorescein and chloride ions were calculated as 81.9 mm^3^ nmol^−1^ and 3.43 × 10^5^ mm^3^ nmol^−1^, respectively (Figure S1). Using these values, *θ_f_* and *θ_Cl_* were determined to be 1.68 × 10^−7^ and nearly 1, respectively. This indicates that in physiological environments (such as in PBS), chlorine ions occupy nearly all available binding sites, thereby limiting fluorescein–gold interaction and permitting relatively unhindered fluorescein transport over large distances (as in the case of in-plane transport).

A comparison of the transport rates in PBS for the three film types revealed the influence of film properties on the in-plane molecular transport (Figure 3c). Linear regression analysis of each film type’s cumulative release profiles was used to extract the release rate, which was equivalent to the in-plane molecular transport rate. Compared to the release rate for np-Au, the coarser film (A-np-Au) did not lead to a statistically different release rate (*p* value = 0.66); however, the film with half the thickness (t-np-Au) resulted in a statistically significant decrease in the release rate (from 1.98 ± 0.70 to 0.69 ± 0.28 ng day^−1^; *p* value = 0.04), as expected from the reduced cross-sectional area for molecular flux. We attributed the slight reduction in A-np-Au’s release rate (from 1.98 ± 0.70 to 1.57 ± 0.84 ng day^−1^) to pore coalescence and shrinkage in film thickness, which effectively reduces the cross-sectional area for molecular flux [43,48]. Steric effects due to narrow pore constrictions were ignored as the hydrodynamic radius of fluorescein (5 Å) is orders of magnitude smaller than the pore radius of np-Au (20–120 nm) [43,60,61]. To rule out any transport along the interface between the metal and the PDMS manifold, we used planar gold (pl-Au) films as a control for both DI water and PBS environments. For both solutions, negligible molecular release (hence transport) was observed for pl-Au (Figure 3d), confirming that fluorescein transport is primarily occurring through the np-Au thin films in the PBS condition.

In order to verify the relationship between the release rate to transport rate, and to determine the effective diffusion coefficients from experimentally obtained released rates, we employed Fick’s 1st Law for porous media [62,63], which is given by

(2) $$n\;=\;\frac{AD_{b}\varepsilon }{\tau }\cdot \frac{\Delta C}{\Delta x}\;=\;AD_{e}\cdot \frac{\Delta C}{\Delta x}$$

where *n* is the molar flux rate (determined in Figure 3a), *A* is the geometric cross-sectional area of the channel (i.e., thin film cross-section), *D_b_* is the diffusion coefficient of fluorescein in bulk solution, *D_e_* is the effective diffusivity, *ε* is the porosity, *τ* the diffusive tortuosity, *C* is the concentration, and *x* is the length of molecular transport region. It is important to note that the effective diffusivity captures the hindrance of transport through a porous media (compared to an unobstructed channel), yet it does not infer information about the actual diffusivity within interconnected pores (nanochannel). Using the right-hand side of Equation 2, we extracted the experimental effective diffusivity (*D_e_*) for each film type (Table 1). These diffusivities are all below the bulk diffusivity of fluorescein (4.2 × 10^−6^ cm^2^ s^−1^) [64], which would be expected due to hindered transport in a nanoporous film [50]. In tandem, we calculated theoretical effective diffusivities by scaling the previously reported bulk diffusivity of fluorescein (*D_b_*) [64] with the porosity and tortuosity values listed in Table 1. The relative errors with respect to the theoretically determined effective diffusivity values are 44%, 55%, and −4% for np-Au, A-np-Au, and t-np-Au respectively. Thicker films have been previous shown to have larger cracks due to relaxation of tensile stress accumulation during dealloying via cracking, which increases the porosity near the surface of the film, and thereby increasing the effective diffusivity [65].

**Figure 4:**
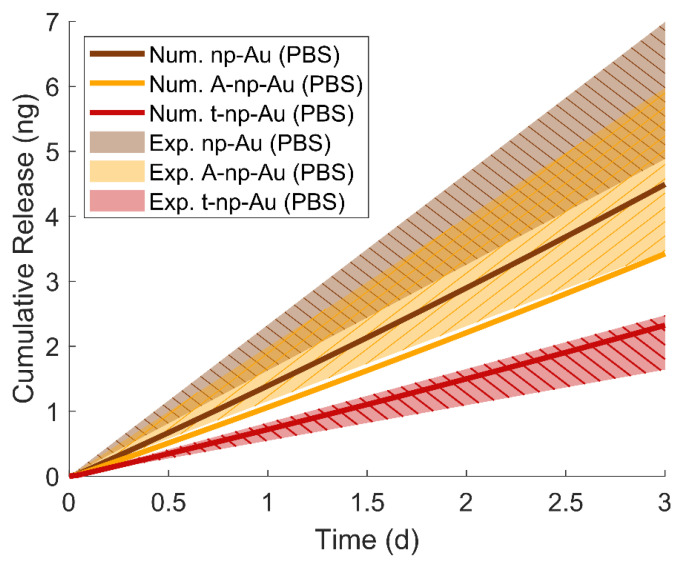
Comparison of the numerical solutions of each film’s molecular transport profiles (solid lines) to the experimental values (shaded region representing one standard error from the mean).

To further verify our experimental results, we used COMSOL Multiphysics 5.5 package to obtain two-dimensional numerical solutions using the Species Transport in the porous media module. The geometry was constructed via three tangent rectangles each having a length of 1 mm and height that corresponds to the thickness of each film type (Figure S2a). For simulating the PBS condition, we assumed that fluorescein-gold interactions are negligible due to the dominant halide–gold interactions, and hence did not consider any adsorption term. However, for the DI water condition, the non-specific fluorescein-gold adsorption must be considered; therefore, we used the calculated values for Langmuir isotherm constant (*K_eq,f_*) and maximum available adsorption sites (*Q_max_*) (Figure S1). Using additional parameters that were previously discussed such as porosity, tortuosity, and bulk diffusivity, we were able to determine time-dependent concentration profiles across the np-Au thin-film (Figure S2b,c). Comparing DI water and PBS simulation results validates the importance of adsorption on the observed in-plane transport rate through np-Au thin-films. For the PBS case, the transport quickly reaches a steady state (Figure S2b), while for the DI water case, the transport does not reach a steady state even after three days. The calculated cumulative release amounts are approximately within a single standard error of the experimental results (Figure 4), indicating a good agreement between the experimentally and numerically obtained transport rates. The simulations underestimate the transport rate for both np-Au and A-np-Au. We attributed this to cracks in the film that may be increasing the transport rate yet that cannot be accurately captured in a simple numerical model.

### 3.3. Biofouling Resilience

**Figure 5:**
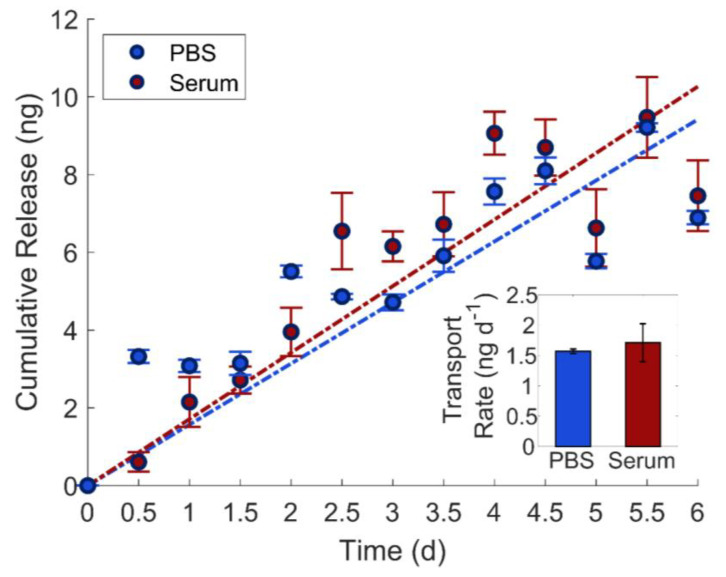
In-plane molecular transport in biofouling conditions. Both the cumulative release profiles and transport rates (inset) show negligible difference between PBS and serum conditions demonstrating biofouling resistance on np-Au thin-films (mean ± SE, *n* = 3).

Biofouling can be a significant impediment to implantable drug delivery systems, especially those with nanostructured delivery components, because the small openings can be blocked by non-specific adsorption of biomolecules such as proteins [66]. Previous studies have shown that np-Au acts as an intrinsic sieve, where partial occlusion of the irregular pores with proteins still allows for small-molecule transport into and out of the nanoporous films [39,67,68]. Here, we assessed the molecular release performance of the in-plane transport device in a biofouling condition. Specifically, we filled the source and sink reservoirs with fluorescein in PBS (Fluo + PBS) and 10% fetal bovine serum (FBS) in PBS (FBS + PBS), respectively. The FBS mimics the rich diversity of proteins in physiological samples. For this study, we only used the np-Au films with the original morphology, as previous studies have shown that np-Au films with larger pores exhibit reduced biofouling resilience, due to increased penetration of biofouling proteins into the pores resulting in higher pore occlusion [39]. We did not observe a statistically significant difference (*p* value = 0.70) between the release rates for release into the protein-rich solution (FBS + PBS) and the control solution (PBS) (Figure 5). This result agrees with the previous studies and highlights that small-molecule release can be maintained even biofouling conditions, which is critical for implantable drug delivery platforms.

### 3.4. Chemical Gating 

**Figure 6:**
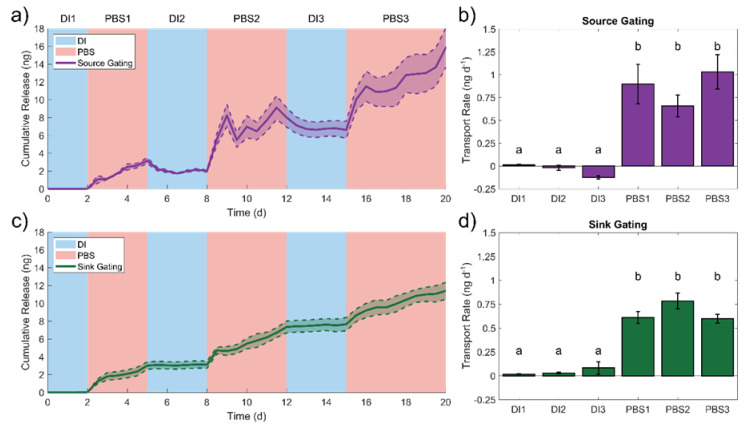
Cumulative release profiles and transport rates demonstrating the chemical gating of fluorescein transport via source (**a**,**b**) and sink (**c**,**d**) gating. The letters indicate statistically significant groups (*p* < 0.05) (mean ± SE), *n* = 6 for sink gating and *n* = 3 for source gating.

We previously discussed the influence of halides on molecular transport in np-Au. Briefly, PBS (halide presence) enabled in-plane molecular transport while DI water stopped the transport. Here, we leveraged this competitive surface adsorption-mediated phenomenon and investigated its potential for chemical gating of fluorescein release. To that end, we tested two different scenarios, in which the ionic environment was changed either only at the source reservoir (source gating) or only at the sink reservoir (sink gating). Specifically, for source gating, we switched the source reservoir content from Fluo + DI to Fluo + PBS while maintaining the sink with DI water. Conversely, for sink gating, we switched the sink reservoir content from DI water to PBS while maintaining the source with Fluo + DI. For both cases, there was a steady-state molecular release when one reservoir contained PBS and there was no release when both reservoirs contained DI water (Figure 6a,c). Additionally, we noted a transient response when switching from DI water to PBS, with an initial non-linear release profile for 12 h, followed by a linear profile for the remainder of the PBS incubation. We hypothesized that the non-linear release was due to desorbed fluorescein ions emptying into the sink reservoir following the switch to the PBS environment. After the initial 12 h, the transport rate reached a steady state, plausibly due to the molecular adsorption/desorption events resolving. When switching back to DI water condition (DI2), the adsorbed chloride ions diffused out of the np-Au network due to the large concentration gradient between the np-Au film and reservoirs. Once the np-Au film was devoid of chloride ions, non-specific adsorption of fluorescein onto the gold surfaces resumed, thereby ceasing molecular transport and stopping fluorescein release into the sink. For both gating scenarios, the release rates determined for each cycle (using linear regression) revealed two different clusters (Figure 6b,d) with statistical significance, as determined by a one-way ANOVA/post-hoc Tukey test (*p* value = 6 × 10^−5^ for source gating and *p* value = 1.14 × 10^−12^ for sink gating). Not only there is a stark difference in the release rate for DI water and PBS conditions, but this difference is highly reproducible across multiple gating cycles. The average release rates for source gating were −0.04 ± 0.07 and 0.86 ± 0.19 ng day^−1^ for the DI water and PBS conditions, respectively. The average release rates for the sink gating were 0.04 ± 0.03 and 0.66 ± 0.10 ng day^−1^. For both gating scenarios, where only one reservoir contains PBS at a time, the release rates were expectedly less than the release rate when both reservoirs contained PBS, which was 1.98 ± 0.70 ng day^−1^ (Figure 3a). These results suggest that the in-plane transport can be gated in response to ionic strength changes in the reservoir content. Put another way, the release rate can be adjusted by modulating the source reservoir contents, which would be located distal to the actual delivery site (sink reservoir). Alternatively, the changes at the site of drug delivery (e.g., tissue) could be used to modulate the release rate. The sensitivity and selectivity of the gold surfaces to the competitive adsorption/desorption (the enabling phenomenon for gated release) can be further tuned by immobilizing functional molecules on the gold surfaces via thiol-gold chemistry, as previously described [42].

## 4. Conclusions

In summary, we studied the influence of ionic environment on in-plane transport of fluorescein ions through np-Au thin films with different morphologies and thicknesses, where the presence of halides enabled molecular transport by reducing non-specific fluorescein (small-molecule drug surrogate) adsorption onto the pore walls. We validated the underlying physics of the in-plane transport and the experimental results with both analytical and numerical (COMSOL Multiphysics® model) methods using an extended Langmuir isotherm model and parameters describing the different np-Au films. The use of the microfluidic device allowed for modeling an anatomical case where the reservoir (source) and the drug delivery site (sink) would be in different locations yet connected with the np-Au thin film for molecular transport. Since np-Au thin films can be patterned via conventional microfabrication techniques, the in-plane transport phenomenon can be translated into multiple electrode arrays [41,69], where np-Au electrode traces can transport small molecules along the traces from the external electrical pads (coupled to distinct pharmaceutical reservoirs) to the individual electrode sites for subsequent molecular release. In this scheme, the pharmaceutical dosing can be precisely controlled by voltage-gated release at the electrode sites [70]. We showed that the molecular release rate can also be modulated (gated) by changing the ionic composition/strength at both the source and sink reservoirs, attaining reproducible gated molecule release across several on/off cycles. The device exhibited sustained molecular release in biofouling conditions, where the irregular pore shape and non-unimodal pore size distribution allowed small-molecule transport despite non-specific protein adsorption onto the pore entrances at the source and sink reservoirs. This size exclusion mechanism can find further utility, where np-Au acts as a microbial filter (achieved by tens of nm pore sizes) separating the source and sink reservoirs. This would be highly useful for both in vitro and in vivo cases, where the source reservoir does not need to be kept sterile, while the sink reservoir can remain sterile for cell culture or tissue contact. This would further allow for using the source reservoir for replenishing pharmaceuticals (via several methods involving hypodermic needle-accessible membranes [2,13,71]) without having the remove the implanted devices. In addition, np-Au’s antimicrobial properties [72], compatibility with biomolecules and tissue [38], and reproducibility in manufacturing [41] fulfill aspects of the Safe-by-Design approach for nanobiomaterial development, which highlights np-Au’s potential for clinical translation [73]. Recent demonstrations of pharmaceutical release from np-Au with biological relevance (e.g., doxorubicin [74], Ara-C [54], and salicylic acid [75]) further support the clinical relevance. Taken together, we expect that the demonstrated phenomenon and the microfluidic model will create new opportunities for biological experiments and fundamental studies of transport phenomena in nanoporous materials.

## Figures and Tables

**Table 1:** Properties for each np-Au film type and the corresponding experimentally- and theoretically-derived effective diffusivities.

Film Type	Porosity, ε	Tortuosity, τ	Exp. Effective Diffusivity (cm^2^ s^−1^)	Theo. Effective Diffusivity (cm^2^ s^−1^)	Relative Error w.r.t Theoretical Diffusivity
np-Au	0.55	1.35	2.46 × 10^−6^	1.71 × 10^−6^	44%
A-np-Au	0.49	1.42	2.22 × 10^−6^	1.44 × 10^−6^	54%
t-np-Au	0.55	1.35	1.65 × 10^−6^	1.71 × 10^−6^	−4%

## Data Availability

The data presented in this study are available on request from the corresponding author.

## Supplementary Materials

The following are available online at https://www.mdpi.com/2079-4991/11/2/498/s1, Table S1: Properties of np-Au and A-np-Au, Figure S1: Langmuir Constant Calculation, Figure S2: COMSOL Simulation.
