# Supplementary material for: Chemically-Gated and Sustained Molecular Transport through Nanoporous Gold Thin Films in Biofouling Conditions
Source: Nanomaterials (Basel). 2021 Feb 16;11(2):498. doi: 10.3390/nano11020498 (PMC7920421; doi:10.3390/nano11020498)
Supplement: Supplementary file 1 [file nanomaterials-11-00498-s001.pdf]

nanomaterials

Supporting Information

Chemically-Gated and Sustained Molecular Transport through Nanoporous Gold Thin Films in Biofouling Conditions

Barath Palanisamy [1], Noah Goshi [1] and Erkin Seker [2,*]

[1] Department of Biomedical Engineering, University of California, Davis, Davis, CA 95616, USA; bpalanisamy@ucdavis.edu ; nkgoshi@ucdavis.edu
[2] Department of Electrical and Computer Engineering, University of California, Davis, Davis, CA 95616, USA; eseker@ucdavis.edu
* Correspondence: eseker@ucdavis.edu ; Tel.: +1-530-752-7300

Volumetric Porosity Calculation for Each Type of Film

The volumetric porosity for np-Au and t-np-Au were assumed to be the same as decreasing the sputtering duration (used for adjusting the film thickness) does not have an influence on alloy composition. We estimated the porosity via "rule of mixtures", where the removal of silver atoms from the alloy will result in a volumetric void proportional to the initial atomic percentage of silver in the alloy since gold and silver have similar lattice constants. Since gold atoms are not removed during dealloying or annealing, the porosity will be inversely related to the volume occupied by the remaining gold atoms, where the total np-Au volume is adjusted to take into account film shrinkage (Table S1), as described previously [1].

Table S1: Summary of the relevant properties of np-Au and A-np-Au thin-films.

	Description	Values
**np-Au**	Length (mm)	1
	Width (mm)	5
	Height or film thickness (nm)	495
	Total volume of np-Au (mm^3)	2.48 x 10^{-3}
	Porosity of np-Au	55%
	Volume of gold in np-Au (mm^3)	1.11 x 10^{-3}
**A-np-Au**	Length (mm)	1
	Width (mm)	5
	Height or film thickness (nm)	437
	Total volume of A-np-Au (mm^3)	2.19 x 10^{-3}
	Pore volume A-np-Au (mm^3)	1.07 x 10^{-3}
	Volumetric porosity of A-np-Au	49%

Langmuir Constant Calculations

To use the extended Langmuir isotherm model, Langmuir constants for both chloride-gold and fluorescein-gold adsorption must be found. $K_{eq,Cl}$ was calculated from previously reported chloride-gold free energy of adsorption (-11.7 kcal mol^{-1}) [2]. The relationship between free energy of adsorption and Langmuir constant is

$$\Delta G = -RT\, ln[\frac{K_{eq,Cl}}{\gamma_e}(1\, mol\, L^{-1})] \quad \text{(S1)}$$

where ΔG is free energy of adsorption, R is the gas constant, and T is absolute temperature, and γ_e is the activity coefficient of chloride ions in PBS [3]. γ_e is calculated using the Debye–Hückel limiting law to yield a value of 0.65. Using Equation S1, $K_{eq,Cl}$ came to be 3.43 x 10^5 mm^3 $nmol^{-1}$.

$K_{eq,f}$ was experimentally found by analyzing data from a separate through-thickness fluorescein release experiment. Following previously reported protocols[1], we varied loading concentration from 1 μM to 10 mM and determined the amount of eluted fluorescein. This allows us to use the linear form of the regular Langmuir isotherm model to find each of the constants

$$\frac{1}{Q} = \frac{1}{K_{eq,f}Q_{max}} * \frac{1}{C} + \frac{1}{Q_{max}} \rightarrow y = mx + b \quad \text{(S2)}$$

where Q refers to amount of absorbed fluorescein ions per mass of gold, Q_{max} refers to maximum number of binding sites per mass of gold, C refers to concentration of fluorescein in void volume or loading concentration. To calculate the amount of adsorbed fluorescein for each loading concentration, we subtracted fluorescein within void volume from total fluorescein released. Dividing this result by the mass of gold in np-Au yields Q, as illustrated in Figure S1. By repeating this procedure for each loading concentration and running linear regression with x-axis as $1/C$ and y-axis as $1/Q$, $K_{eq,f}$ was determined to be 81.9 mm^3 $nmol^{-1}$ and Q_{max} was 1060 nmol g^{-1}.

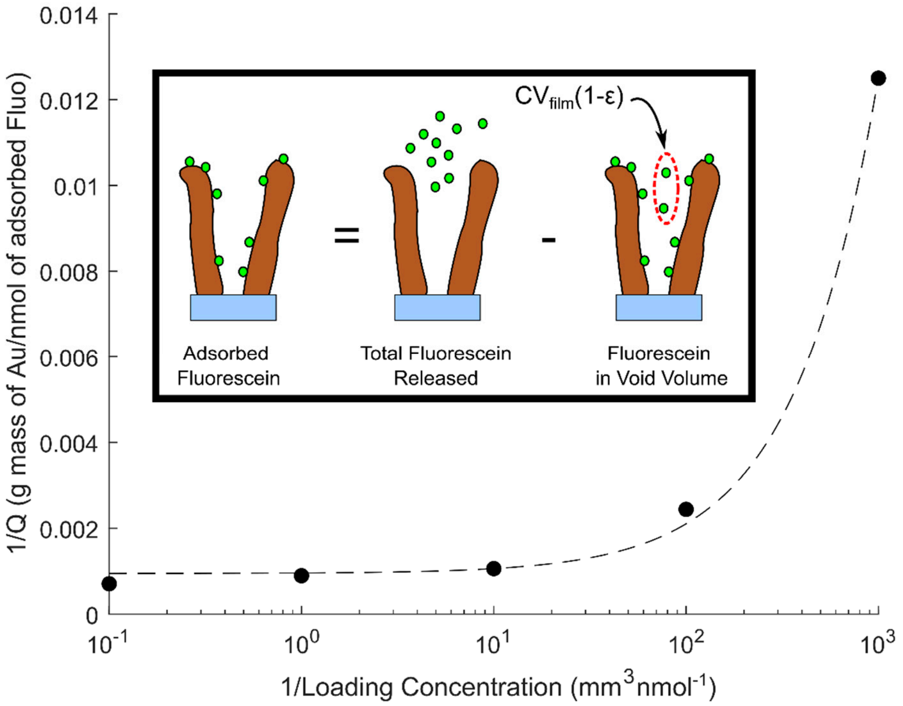

Figure S1: To calculate $K_{eq,f}$, a linear form of the regular Langmuir isotherm adsorption model was used. $K_{e,f}$ can be extracted by running linear regression on varying loading concentrations and its respective adsorbed amounts. Adsorbed fluorescein can be determined by subtracting fluorescein in void volume from total fluorescein released. The latter can be calculated by the equation listed above where C represents loading concentration, V_{film} represents volume of thin film, and ε represents porosity of np-Au (inset). Q_{max} was determined to be 1060 nmol g^{-1} and $K_{eq,f}$ was 81.9 mm^3 $nmol^{-1}$.

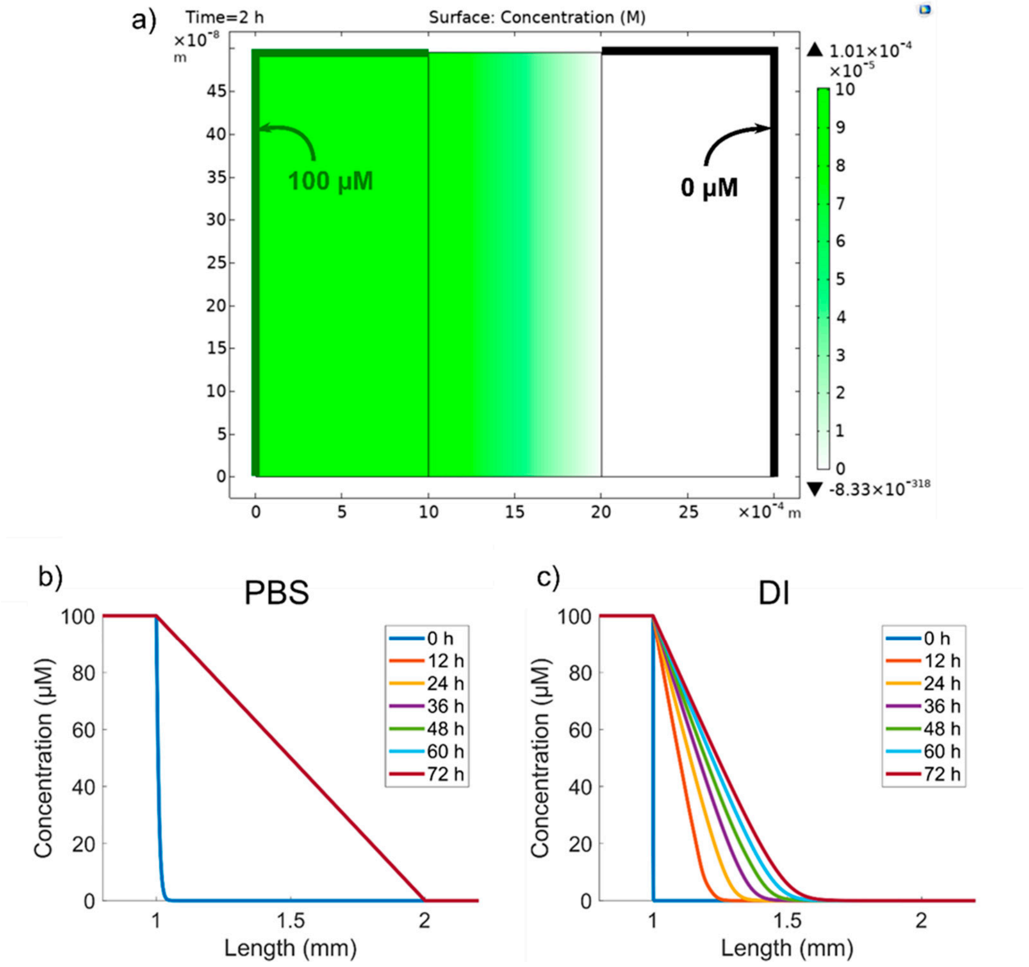

Figure S2: Using COMSOL Multiphysics® 5.5, a two-dimensional numerical solution for in-plane transport of fluorescein through np-Au was found using the Species Transport in Porous Media module. (a) A side-view of the np-Au was constructed via three rectangles. As indicated, concentration boundary conditions were set at opposite edges of the film. The magnitude of green in the 2D plot represents concentration of fluorescein. (b) Visualizing the concentration line profiles, we see that the PBS condition reaches steady state at 12 h, as any line profiles after 0 h overlap with each other. (c) The line profiles for the DI water condition indicate that even after 72 h, transport has not reached steady state.

References

1. Kurtulus, O.; Daggumati, P.; Seker, E. Molecular release from patterned nanoporous gold thin films. *Nanoscale* **2014**, *6*, 7062–7071, doi:10.1039/c4nr01288g.
2. Bodé, D.D. Calculated free energies of adsorption of halide and hydroxide ions by mercury, silver, and gold electrodes. *J. Phys. Chem.* **1972**, *76*, 2915–2919, doi:10.1021/j100664a022.
3. Liu, Y. Is the free energy change of adsorption correctly calculated? *J. Chem. Eng. Data* **2009**, *54*, 1981–1985, doi:10.1021/je800661q.
